# Doubly Uniparental Inheritance of Mitochondria As a Model System for Studying Germ Line Formation

**DOI:** 10.1371/journal.pone.0028194

**Published:** 2011-11-29

**Authors:** Liliana Milani, Fabrizio Ghiselli, Maria Gabriella Maurizii, Marco Passamonti

**Affiliations:** Department of Biologia Evoluzionistica Sperimentale, University of Bologna, Bologna, Italy; Harvard Medical School, United States of America

## Abstract

**Background:**

Doubly Uniparental Inheritance (DUI) of mitochondria occurs when both mothers and fathers are capable of transmitting mitochondria to their offspring, in contrast to the typical Strictly Maternal Inheritance (SMI). DUI was found in some bivalve molluscs, in which two mitochondrial genomes are inherited, one through eggs, the other through sperm. During male embryo development, spermatozoon mitochondria aggregate in proximity of the first cleavage furrow and end up in the primordial germ cells, while they are dispersed in female embryos.

**Methodology/Principal Findings:**

We used MitoTracker, microtubule staining and transmission electron microscopy to examine the mechanisms of this unusual distribution of sperm mitochondria in the DUI species *Ruditapes philippinarum*. Our results suggest that in male embryos the midbody deriving from the mitotic spindle of the first division concurs in positioning the aggregate of sperm mitochondria. Furthermore, an immunocytochemical analysis showed that the germ line determinant Vasa segregates close to the first cleavage furrow.

**Conclusions/Significance:**

In DUI male embryos, spermatozoon mitochondria aggregate in a stable area on the animal-vegetal axis: in organisms with spiral segmentation this zone is not involved in cleavage, so the aggregation is maintained. Moreover, sperm mitochondria reach the same embryonic area in which also germ plasm is transferred. In 2-blastomere embryos, the segregation of sperm mitochondria in the same region with Vasa suggests their contribution in male germ line formation. In DUI male embryos, M-type mitochondria must be recognized by egg factors to be actively transferred in the germ line, where they become dominant replacing the Balbiani body mitochondria. The typical features of germ line assembly point to a common biological mechanism shared by DUI and SMI organisms. Although the molecular dynamics of the segregation of sperm mitochondria in DUI species are unknown, they could be a variation of the mechanism regulating the mitochondrial bottleneck in all metazoans.

## Introduction

The origin of Primordial Germ Cells (PGCs) is a central issue in developmental biology. In many animals, cells that inherit the germ plasm during the first embryonic divisions give rise to PGCs [1]–[8]. The germ plasm is a specific part of the egg cytoplasm, whose composition is similar in widely divergent phyla: it mainly contains proteins from conserved germ cell-specific genes and RNAs, mRNAs and non-coding RNAs, used for translation when post-transcriptional processing reactivates after fertilization [9]–[11]. A typical component of germ plasm is the DEAD-box RNA helicase Vasa, firstly characterized in *Drosophila melanogaster* [12], [13], and then isolated in many invertebrates and vertebrates [14]. Inactivation of this helicase suppress the formation of PGCs [15]–[18]. Interestingly, the aggregation of *vasa* products in the germ plasm appears to occur either at protein or transcript level, depending on the organism [19]. Germ plasm can be identified with Transmission Electron Microscopy (TEM) by the presence of electron-dense granulofibrillar material which is called “nuage”, often positioned near the nucleus and associated with mitochondria [7], [10], [20]. The nuage stores RNAs and proteins that are produced early in oogenesis and, in oocytes, it associates with a distinctive structure known as Balbiani Body (Bb), which includes also a mitochondrial mass and Golgi complexes [7], [10], [21]. Subsequently, the Bb is transported in the cytoplasm area of the egg that, after fertilization, is taken up by PGCs [10]. In male germinal cells, the nuage is called chromatoid body (Cb), and is typically associated with mitochondria [22], [23]. The Cb is first clearly seen in mid- and late pachytene spermatocytes as an inter-mitochondrial dense material [23]. Then, during spermiogenesis, the Cb acquires a lobular structure and is surrounded by a multitude of vesicles, near the Golgi complex, and frequently connected by material continuities with the nucleus through nuclear pores [23]–[25]. Later in spermiogenesis, at least in mammals, the Cb takes part in the formation of the residual body, a ring adjacent to the midpiece [23], [26].

The frequent colocalization of mitochondria and nuage suggests a connection between these organelles and germ line development. For example, mitochondrial ribosomes are found extramitochondrially and are required to produce proteins necessary for germ cell formation [4], [27]–[32]. Moreover, evidence from TEM analysis documented the extrusion of material and cristae from mitochondria forming nuage both in gametes during gametogenesis [20], [33] and in developing embryos [20], [32], [34]. Actually, in early embryos a mitochondrial-type translation is required for germ cell formation, which is disrupted by the injection of prokaryotic translation inhibitors [35]. Another supporting clue to a role of mitochondria in germ line formation comes from their origin: they have a common ancestor with the endosymbiont Rickettsiales [36], some of which are known to distort the sex of their host. *Wolbachia*, for example, induces male killing, feminization, parthenogenesis, cytoplasmic incompatibility [37], [38], and methylation pattern modification in gonads [39]. Accordingly, depletion of mtDNA results in significant changes in the methylation pattern of a number of nuclear genes, some of which are reversed by the restoration of mtDNA [40].

Another fundamental question in developmental biology is which mitochondria effectively enter the germ line. A bottleneck has been proposed based on the observation that mitochondrial genomes in each individual are largely homoplasmic, despite their high mutation rate [41]–[43]. In oocytes of many organisms two populations of mitochondria can be distinguished: one population is aggregated in the Bb and enters the embryo PGCs during development [7], [21], [34], while the remaining mitochondria spread in the oocyte and are transmitted to the somatic lineage [44]–[46]. For this reason, the Bb mitochondrial population or a part of it may actually represent the mitochondrial bottleneck [47]. Since all mitochondria are usually transmitted to embryos through oocytes, the bottleneck is generally believed to be under female control, but factors from spermatozoon might also be involved in mitochondrial inheritance and selection [48]. The process by which mitochondria enter the developing germ line is still unknown, but the involvement of microtubules in their general distribution was confirmed by experiments using inhibitors to microtubule polymerization [49]–[51].

To address the relationship between mitochondria and germ line formation we study an unusual system of mitochondrial transmission known as Doubly Uniparental Inheritance (DUI) (reviewed in [52], [53]). Commonly, metazoan mitochondria experience Strictly Maternal Inheritance (SMI), i.e. they are transmitted by females only, because mitochondria from spermatozoon are excluded or degraded [54]–[57]. The only known exception comes from some bivalve molluscs ([58] and references therein), in which two mitochondrial lineages are inherited, one through eggs (F-type), the other through sperm (M-type), and showing up to 52% sequence divergence [59]. In DUI male embryos, spermatozoon mitochondria form an aggregate [49], [60]–[62], and, during development, this aggregate enters PGCs, as proved by the specific presence of M-type mtDNA in spermatozoa [63], [64]. In DUI female embryos sperm mitochondria are dispersed and the mitochondrial inheritance would be as in SMI species [49], [60], [61], [64]–[66]. Moreover, in DUI *Mytilus* species sex-ratio distortion was observed: females were found producing a female-biased offspring and showing a majority of dispersed patters, other females produced a male-biased progeny with a majority of aggregated patters, and others a 50∶50 sex-ratio, with a balanced frequency of the two patterns [61], [65], [67]. Recently, sex-ratio biased progenies were also found in the DUI species *Ruditapes philippinarum* Adams & Reeve [68]. The peculiar segregation pattern of spermatozoon mitochondria, correlated with the existence of sex-biased lineages, led to the hypothesis that M-type mitochondria could have an active role in the masculinization of the gonad, achieved through a series of specific signals between the nucleus and mitochondria [67].

The Manila clam *R. philippinarum* belongs to the family Veneridae, it is strictly gonochoric and its gonad forms every year at the beginning of the mating season, after which it is degraded [69]. During the non-reproductive season sex cannot be determined [69]. Like other protostomes, molluscs follow a spiralian embryonic development: embryo blastomeres receive a specific content of cytoplasm, and they can be tracked through development up to the formation of organs [70]. PGCs arise from the 4d mesentoblast formed during the sixth cell division ([70] and references therein), but the mechanisms of sexual differentiation of the gonad are still unknown.

Exploiting the peculiar features of DUI, we investigated the specific segregation of spermatozoon mitochondria in clam embryos. Although the molecular dynamics of their segregation is still largely unknown, it could be a variation of the mechanism that regulates the mitochondrial bottleneck in all metazoans. In *R. philippinarum*, we found a series of typical structures related to germ line formation, pointing to a common biological mechanism shared by DUI and SMI organisms. All that considered, DUI can certainly provide important information on mitochondrial inheritance dynamics and functions.

## Methods

### Spawning induction and fertilization

*R. philippinarum* clams used in mating experiments were collected in Goro (Italy) during the reproductive season (July/August). The spawning was induced with cycles of cold and warm artificial sea water (e.g. reverse osmosis water added with RedSea Coral Pro aquariology sea salt, at 22 and 29°C) every 30 min. After fertilization, embryo development was visually checked with an optical microscope. Samples of 2-, 4-, 8-blastomere embryos were collected. Clams for gonad analysis were sampled in February (developing), and in May (mature).

### MitoTracker staining

The vital mitochondrial dye MitoTracker Green FM (Molecular Probes), dissolved in anhydrous dimethylsulfoxide (DMSO) (Sigma), was added to the sperm suspension at 200 nM of final concentration. The sperm was incubated for 20 minutes at room temperature (RT) in the dark and then used in small amount to fertilize eggs. The embryos were put on slides coated with 3-aminopropyltriethoxysilane (APES; Sigma) and mounted directly in sea water. The visualization was performed by a Nikon Eclipse 90i fluorescence microscope using a FITC filter and pictures were recorded by NIS-elements AR 3.0 software.

### Transmission Electron Microscopy (TEM)

Sample preparation for TEM analysis was performed as follows: gonadic tissue was dissected with a scalpel and immediately fixed for 2 hours in glutaraldheyde 2%, then rinsed in salt water and post-fixed in 1% osmium tetroxide in salt water for 1 h. After rinsing in salt water, we proceeded with dehydration with increasing concentrations of acetone (50–100; 70% acetone solution contained 1% uranyl acetate). Samples were then embedded in resin (Fluka Durcupan ACM). Polymerization was completed after 3–4 days at 60°C, followed by 1–2 days at 45°C. A Reichert ultramicrotome was used to cut silver-gold ultrathin sections (60–90 nm) using diamond knives. Ultrathin sections were collected on 75 mesh, copper grids coated by formvar and then stained with 3% uranyl acetate and with lead citrate. Sections were examined using a Philips CM100 (PW6021) TEM at 80 kV. Pictures were recorded with a Kodak MEGAPLUS Camera, Model 1.6i, using AnalySIS® Software.

### SDS-PAGE and western blotting

Pieces of gonads and embryos were frozen in liquid nitrogen, stored at −80°C, and then processed by SDS-PAGE (Sodium Dodecyl Sulphate - PolyAcrylamide Gel Electrophoresis) and western blotting. Embryos of 2, 4 and 8 blastomeres were pooled for this analysis. Samples were homogenized in a buffer containing 10 mM Tris-HCl, pH 7.5, 1 mM ethylene glycol-bis(2-aminoethyl ether)-N,N,N',N'-tetraacetic acid (EGTA), 0.1% SDS in the presence of 2x protease inhibitor cocktail tablets (Complete Mini, Roche) and 1 mM PMSF, utilizing an Ultra Turrax T25 Janke & Kunkel IKA-labortechnik. Then samples were centrifuged at 10,000 rpm for 10 minutes at 4°C. The supernatant was stored at −80°C. Gonadic and embryonic extracts of *R. philippinarum* (10 µg) were separated in 8.5% SDS polyacrylamide gels according to Laemmli [71]. For immunoblotting, proteins were transferred to Hybond-ECL membrane (Amersham International, Buckinghamshire, UK). Non-specific protein-binding sites were blocked with 5% dried skimmed milk (Bio-Rad Laboratories, Hercules, CA, USA), 3% Bovine Serum Albumin (BSA), and 0.1% Tween-20 (Sigma) in TBS overnight at 4°C and subsequently washed with 0.1% Tween TBS. To recognize Vasa protein, we utilized an antiserum against chicken Vasa-homolog produced in rabbit already in use in our laboratory (anti-Cvh; [72]). The membranes were treated as described in Maurizii et al. [73]. A bidimensional SDS-PAGE of gonadic extract was carried out as in GE Healthcare Handbook (80-6429-60AC).

### *vasa*-homolog

We analyzed the *vasa*-like gene of *R. philippinarum* found by whole transcriptome sequencing of gonads [68]. An ORF of 2,373 bp was identified, translated and aligned with Cvh (*Gallus gallus*, GenBank BAB12337.1) using T-COFFEE, version 8.99 [74]. Conserved domains were identified using InterProScan, version 4.8 [75].

### Tissue processing and immunocytochemical analyses

#### Embryos

Embryos were fixed in a solution containing 3.7% paraformaldehyde, 0.1% glutaraldehyde, and 1.5 µM taxol in K-PIPES buffer [80 mM K Pipes; 1 mM MgCl_2_; 5 mM EGTA; 0.2% Triton X-100] (pH 6.8) for 30 minutes at 37°C. After several washes in Tris-Buffered Saline solution [TBS: 155 mM NaCl; 10 mM Tris-HCl] (pH 7.4), embryos were put in methanol and stored at 4°C. After rehydration with TBS (pH 7.4), fixed embryos were put on slides coated with APES (Sigma), treated with 50 mM sodium borohydride in TBS for 60 minutes at RT and rinsed in TBS with several changes. Embryos for microtubule staining were digested with 0.01% Pronase E (Merck) in Phosphate-Buffered Saline solution (PBS) [128 mM NaCl; 2 mM KCl; 8 mM Na_2_HPO_4_.2H_2_O; 2 mM KH_2_PO_4_] (pH 7.2), for 6–7 minutes at RT. Permeabilization was performed adding TBS-Triton 1% to all the samples and leaving over night at 4°C.

#### Vasa immunostaining

Non-specific protein-binding sites were blocked with a buffer containing 10% Normal Goat Serum (NGS) and 1% BSA in TBS 0.1% Triton (pH 7.4). The primary antibody (anti-Cvh) was diluted 1∶ 2,000 with TBS containing 0.1% Triton, 10% Normal Goat Serum (NGS), and 1% BSA (pH 7.4) for 48 hours at 4°C. As secondary antibody we employed a goat anti-rabbit polyclonal antibody, conjugated with N,N'-(dipropyl)-tetramethylindocarbocyanine (Cy3) (Zymed, Molecular Probes), diluted 1∶ 300 with TBS containing 0.1% Triton, 10% NGS, and 1% BSA (pH 7.4) for 30 hours at 4°C.

#### Microtubule immunostaining

Embryos were incubated with a monoclonal anti-α-tubulin, clone DM 1A (Sigma), diluted 1∶ 1,800 with TBS-Triton 0.1% and 2% BSA (pH 7.4) for 48 hours at 4°C. Bound primary antibody was detected using Cy3-conjugate anti-mouse, produced in sheep (Sigma), diluted 1∶ 300 with TBS-Triton 0.1%, 2% BSA (pH 7.4), with about 40 hours of incubation at 4°C.

Nuclei of all samples were stained with 1 mM TO-PRO3 (Molecular Probes) diluted 1∶ 2,000 in PBS (pH 7.2) at RT. Samples were mounted in 2.5% 1,4-diazabicyclo[2.2.2]octane (DABCO; Sigma), 50 mM Tris (pH 8) and 90% glycerol, and stored at 4°C.

Controls were performed using embryos in which the first or the second antibody was omitted, and embryos treated only with normal serum. Imaging of embryos was recorded by a confocal laser scanning microscope (Leica confocal SP2 microscope), using Leica software.

### Gonads

Pieces of gonads were removed, fixed in 3.7% paraformaldehyde and 0.1% glutaraldehyde solution in K-PIPES buffer (pH 7) for 3 hours and embedded in 7% agar. Sections of about 100 µm thickness were made using a Lancer Vibratome Series 1000 and post-fixed with increasing concentrations of methanol. Sections were rehydrated and then treated 1 hour and 15 minutes with sodium borohydride 70 mM in TBS (pH 7.4) at RT. After rinsing 1 hour and 15 minutes in TBS-Triton 0.1%, samples were permeabilized adding TBS-Triton 1% and left over night at 4°C. See *Vasa immunostaining* above for Vasa immunolocalization.

## Results

### Gonad morphology and gamete ultrastructure analysis

**Figure 1:**
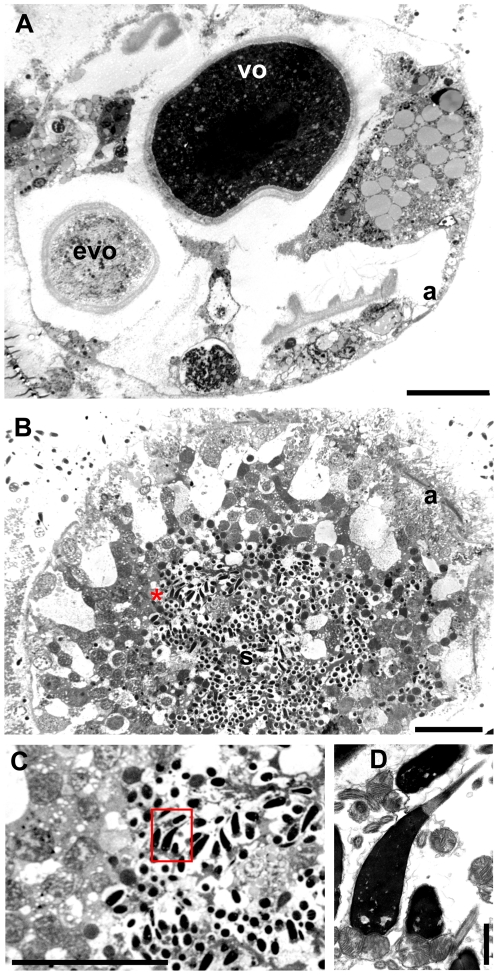
Morphological analysis of female and male gonads in *R. philippinarum* (TEM). (A) A female acinus, with oocytes at different stages of differentiation; evo – early vitellogenic oocyte; vo – vitellogenic oocyte; a – acinus wall (20 µm bar). (B) Portion of a male acinus in which spermatogenesis is developing centripetally, with mature spermatozoa (s) free within the acinus lumen (20 µm bar). (C) Magnification, from B, of the detail at the right side of the red asterisk, showing mature spermatozoa (20 µm bar). (D) The detail squared in C: a sperm head with acrosome and mitochondrial midpiece (1 µm bar).

**Figure 2:**
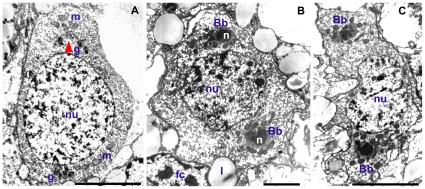
Ultrastructural analysis of the Balbiani body formation in *R. philippinarum* (TEM). (A) Oogonium showing electrondense granules (g) close to two mitochondrial masses (m) at opposite sides of the nucleus (nu) (5 µm bar). (B, C) In early oocytes two identical Balbiani bodies (Bbs) are present, both containing a large and dense nuage (n) and a mitochondrial mass. fc – follicle cell; l – lipid droplet (B: 2 µm bar; C: 5 µm bar).

**Figure 3:**
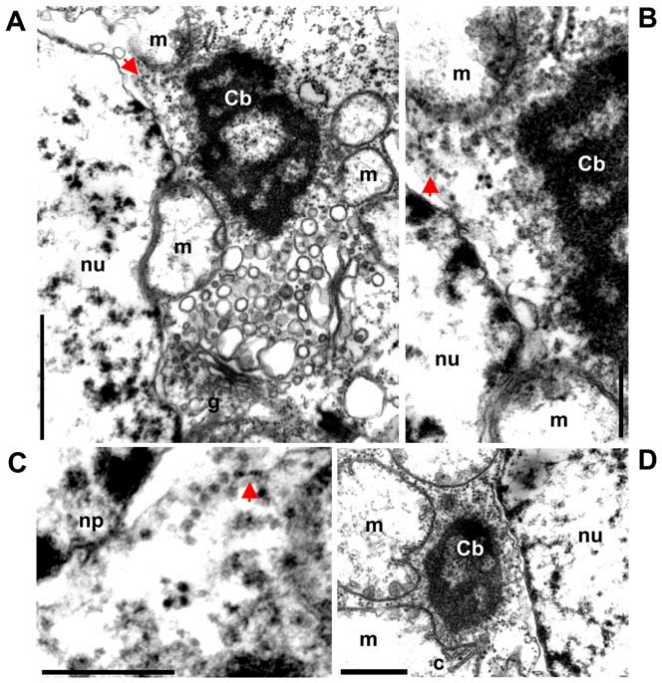
Ultrastructural analysis of the Chromatoid body formation in *R. philippinarum* (TEM). (A) Cytoplasmic area around the Chromatoid body (Cb) in an early spermatid (1 µm bar); nu – nucleus; g – Golgi apparatus. (B) A detail from A showing the region among nucleus, mitochondria (m) and the Cb (250 nm bar). (C) A detail of B: nuclear material outflows from a nuclear pore (np) and a polyribosome formed by ribosomes of two different sizes is visible, the smallest are indicated by an arrow (250 nm bar). (D) The Cb is located close to the nuclear membrane and surrounded by empty mitochondria, some of which showing extruded cristae (c) (500 nm bar). arrow – polyribosome.

Female and male mature gonads of *R. philippinarum* consist of acini made of germinative epithelium supported by connective tissue [69]. Different stages of gamete maturation within a single acinus can be seen ([69]; Figure 1A and B). In female acini, oocytes are grouped at the periphery. During their maturation, oocytes become pedunculated and, shortly before spawning, lose their peduncles and fill the lumen of the acini ([69] and Figure 1A). Spermatogenesis occurs centripetally with mature spermatozoa free and mobile within the lumen of the acini ([69] and Figure 1B–D). Four mitochondria of about 1 µm in diameter form the midpiece of *R. philippinarum* spermatozoon, while egg mitochondria are quite smaller (about 0.6 µm in vitellogenic oocytes) [62]. The ultrastructural analysis of developing gametes allowed the identification of both Balbiani body (Bb, Figure 2) and Chromatoid body (Cb, Figure 3), in early oocytes and early spermatids, respectively. Oogonia showed electrondense granules aggregating near two mitochondrial masses at opposite sides of the nucleus (Figure 2A), where in early oocytes they formed the nuage of two identical Bbs (Figure 2B and C). Each Bb was characterized by a dense nuage of about 1 µm and a mitochondrial mass of 10–28 organelles per section (Figure 2B and C). In early spermatids the ultrastructural analysis allowed the identification of a typical Chromatoid body (Cb) (Figure 3), a bit longer than 1 µm, always located close to the nuclear membrane and surrounded by empty mitochondria (Figure 3). A pore in the nuclear membrane could be seen emitting material near the Cb (Figure 3A–C). In the area among the nuclear pore, the empty mitochondria and the Cb, ribosomes of two different sizes were found on polyribosomes, with the small ones localized in a restricted zone (Figure 3B and C).

### *R. philippinarum vasa*-homolog

**Figure 4:**
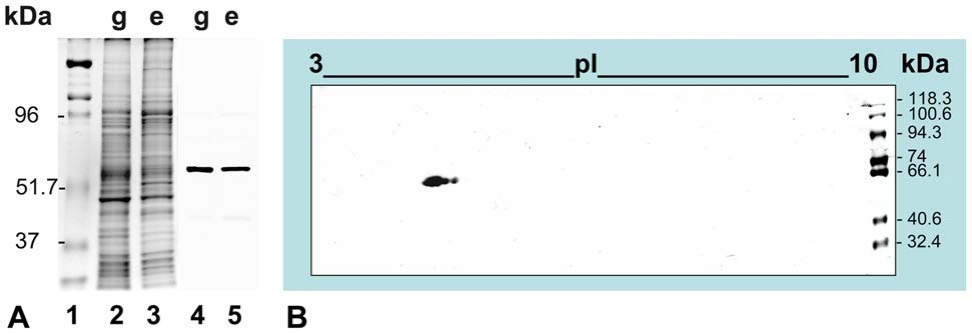
Specificity of the anti-Cvh antibody. (A) Monodimensional electrophoresis and immunoblotting of gonadic and embryonic extracts of *R. philippinarum*. Gel Coomassie stained (lanes 1–3). Lane 1: molecular mass standards with the value in kilodaltons (kDa) on the left. Lane 2: gonadic extract (g). Lane 3: embryonic extract (e). The antiserum against chicken Vasa-homolog (anti-Cvh) marks one protein band of about 65 kDa, both in gonadic and embryonic extracts (lanes 4, 5). (B) Bidimensional immunoblotting of gonadic extract with anti-Cvh. The antibody detects one big spot flanked by two smaller, all of about 65 kDa as molecular mass and with isoelectric point (pI) around 5.

**Figure 5:**
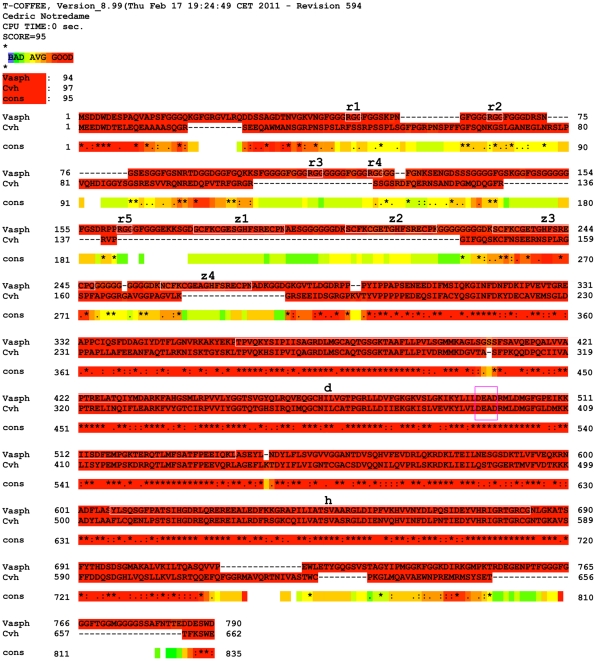
Vasa alignment. T-COFFEE alignment of Vasph (Vasa philippinarum homolog) and Cvh (chicken Vasa-homolog, *Gallus gallus*, GenBank BAB12337.1). Conserved domains are boxed in white and the DEAD motif in pink (see also Figure 6). cons = consensus.

**Figure 6:**
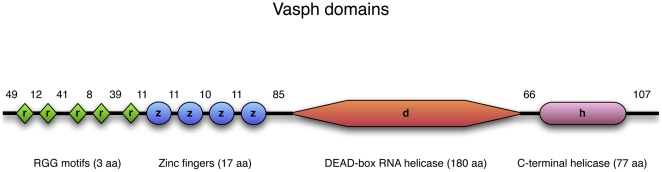
Vasph domains. Vasa conserved domains found in Vasph inferred sequence by InterProScan (version 4.8). There are four CCHC-type zinc fingers (17 aminoacids, aa, each), a DEAD-box RNA helicase (180 aa), and a C-terminal helicase (77 aa). In addition, five RNA-binding RGG motifs are included in the N-terminal region. The number of aminoacids of the regions between the domains are reported above the scheme.

The specificity of the anti-vasa Chicken antibody (anti-Cvh) was tested by performing mono and bidimensional western blot. In both gonad and embryo samples, monodimensional immunoblotting with anti-Cvh detected only one band of about 65 kDa as molecular mass (Figure 4A). In gonadic extract, the bidimensional analysis showed one big spot flanked by two smaller, all of about 65 kDa and isoelectric point (pI) around 5 (Figure 4B). We also analyzed the *vasa* homolog of *R. philippinarum* found by whole transcriptome sequencing of gonads [68]. We named it *vasph* (*vasa philippinarum* homolog). The inferred aminoacid sequence included 790 residues. The T-COFFEE protein alignment with the chicken Vasa-homolog showed a good CORE index (score = 95) [76] (Figure 5). Using InterProScan, four CCHC-type zinc fingers (GO:0008270, location: 176–192; 204–220; 231–247; 259–275), a DEAD-box RNA helicase (GO:0008026, location: 361–540), and a C-terminal helicase (GO:0004386, location: 607–683) were found (Figure 6). In addition, in the predicted protein sequence five RNA-binding RGG motifs were included in the N-terminal region (Figure 6).

**Figure 7:**
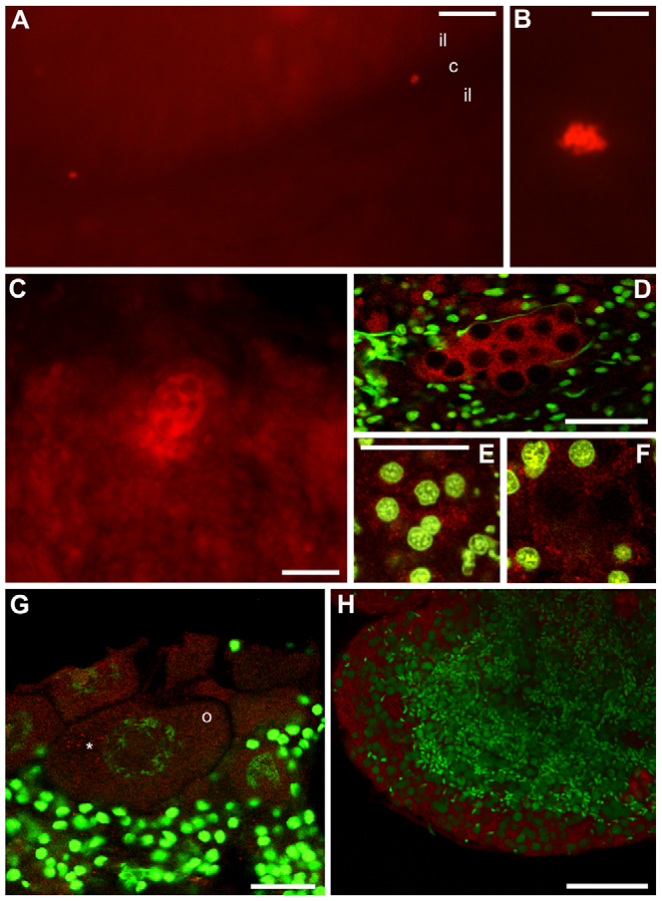
Vasph immunolocalization with anti-Cvh during *R. philippinarum* gonad differentiation. (A–C) Fluorescence microscope analysis on sections of a gonad at the beginning of its differentiation. (A) Two small spots result labeled in the connective tissue (c) between two intestinal loops (il), that are not labeled (250 µm bar). (B) In a magnification of A the staining of the early gonad is better visible (50 µm bar). (C) A following stage of gonadic development, in which the germ cells, strongly stained, start forming acini with some big cavities in the middle (50 µm bar). (D–F) Confocal analysis on sections of a more differentiated gonad. Anti-Cvh (red) and TO-PRO-3 iodide staining DNA (green). (D) Incipient acini with some Vasph stained cells visible on a line around the cavities; the numerous cells of the surrounding connective tissue are not labeled (50 µm bar). (E, F) Two images at various depths, within the same sample of D. (E) Germ cells with big nuclei and a strong Vasph-staining are visible (24 µm bar). (F) In a deeper region of the gonadic tissue observed in E, the labeled germ cells are located around a still empty acinus lumen (same magnification of E). (G, H) Confocal analysis on sections of female and male mature gonads. (G) Oocytes strictly packed in an enlongated acinus show a diffuse cytoplasmic immunolocalization of Vasph. In the biggest oocyte (o), some strongly labeled granules are seen in a region of the cytoplasm (asterisk) (30 µm bar). (H) Male acinus full of spermatozoa: a diffused staining is present only in the spermatogenic cells located near the acinus wall (50 µm bar).

**Figure 8:**
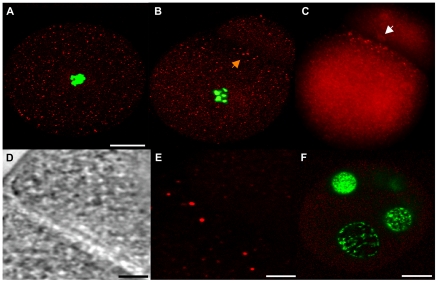
Vasph immunolocalization with anti-Cvh in *R. philippinarum* embryos. (A) Stained egg at confocal microscope showing small granules equally dispersed in the cytoplasm (20 µm bar). (B) Confocal microscope analysis of a 2-blastomere embryo: some small stained spots are dispersed in the cytoplasm, but an aggregation of bigger granules with a stronger labeling is localized close to the cleavage furrow (arrow). Anti-Cvh and TO-PRO-3 iodide staining DNA are represented by red and green, respectively. (C) The same embryo under fluorescence microscope; a trace of the midbody can be perceived (white arrow) (B, C: same magnification of A). (D, E) The same detail of the cleavage shown in B, with transmitted light (D) and confocal microscope (E) (D, E: 4 µm bar). (F) Confocal microscope analysis of a 4-blastomere embryo, in which only secondary antibody was used. No staining is visible (20 µm bar).

Once verified the specificity of anti-Cvh, we analyzed Vasph expression in gonads and in early embryos of *R. philippinarum*. At the beginning of the reproductive season, when gonads start forming, we found that anti-Cvh labeled small groups of cells (Figure 7A and B). These Vasph-stained cells were detected in the connective tissue among unlabeled intestinal loops (Figure 7A), which is the normal position of bivalve gonad formation [69]. In a following stage of gonad differentiation, Vasph-labeled cells formed an empty space in the middle of the aggregate, becoming the wall of incipient acini with a lumen at the center (Figure 7C–F). Finally, in mature gonads acini appeared full of gametes (Figure 7G–H). In a female acinus (Figure 7G), oocytes showed a diffused cytoplasmic immunolocalization of Vasph. A small aggregation of granules was evident in the cytoplasm of the biggest oocyte (Figure 7G). In male acini, a diffused staining was found in spermatogenic cells along the acinus wall (Figure 7H). In spawned eggs, Vasph-staining showed granules dispersed in the cytoplasm, with no evident pattern of aggregation (Figure 8A). On the contrary, in 2-blastomere embryos Vasph immunostaining showed a more strongly stained zone along the cleavage furrow, both with confocal (Figure 8B) and fluorescence (Figure 8C) microscope. Some bigger labeled spots were aggregated where the midbody formed (Figure 8B–E). Control embryos did not show any labeling (Figure 8F).

### Distribution of microtubules and spermatozoon mitochondria in early embryos

**Figure 9:**
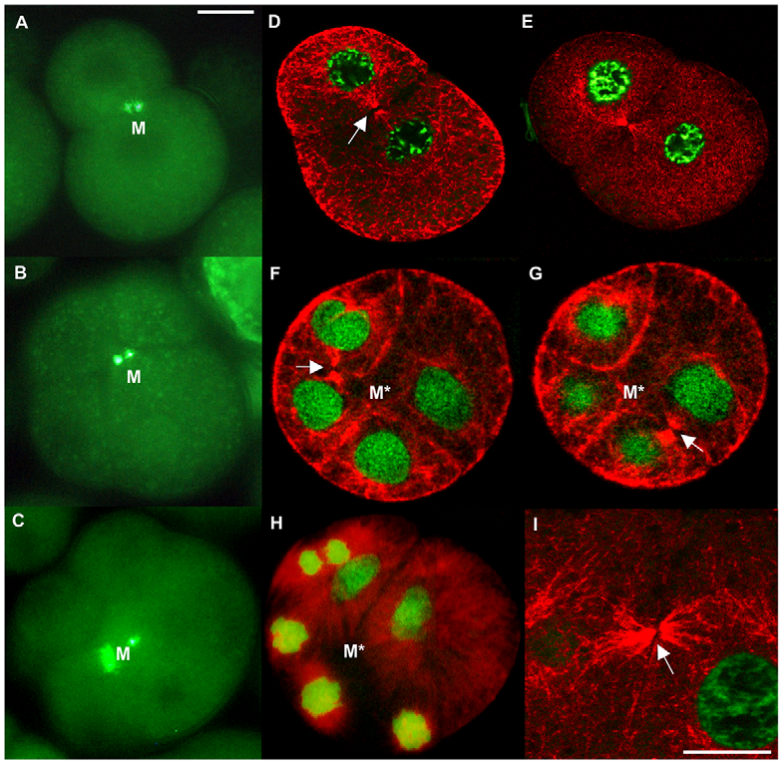
Distribution patterns of spermatozoon mitochondria and microtubules in *R. philippinarum* male early embryos. (A–C) Fluorescence microscope analysis. The staining with MitoTracker Green shows an aggregation of spermatozoon mitochondria (M, in light green) in 2- (A), 4- (B) and 8- (C) blastomere male embryos. (D–I) Confocal microscope analysis. (D, E) Microtubule immunolocalization in 2-blastomere male embryos. Anti-α-tubulin (red) and TO-PRO-3 iodide staining DNA (green). A well defined midbody (white arrow) is localized in the middle of the first cleavage furrow, corresponding to the area in which the four spermatozoon mitochondria aggregate in male embryos. (F, G) Microtubule immunolocalization in 4-blastomere male embryos. The two images are acquired at various depths within the same embryo: the two new spindles to form the 4-blastomere embryo are positioned in tangential directions, far from the region (M*) where the four M-type mitochondria localize (white arrows indicate the midbodies), as clearly shown also in 8-blastomere embryos (H) (bar 20 µm). (I) Detail of a midbody (white arrow) (bar 15 µm).

**Figure 10:**
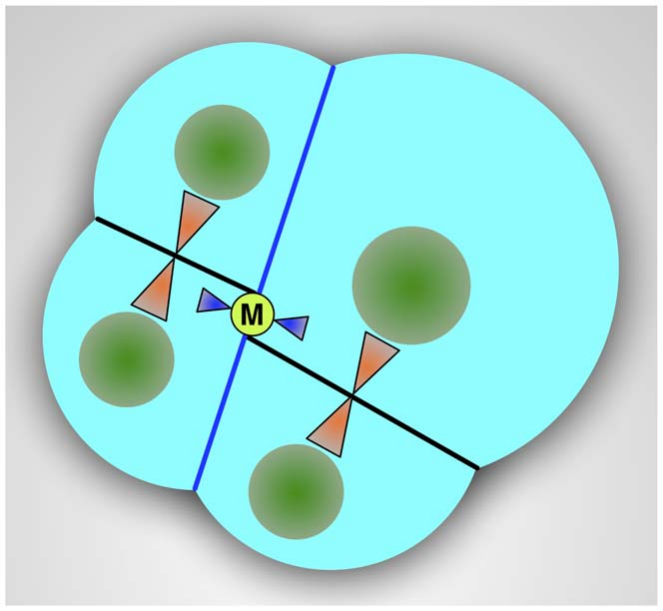
Segregation scheme of spermatozoon mitochondria in DUI male embryos. The scheme shows the position of the two midbodies (orange triangles) in a developing 4-blastomere embryo. The plane of the first cleavage is shown with a blue line, and the first midbody, by this time disassembled, is in blue. During the following divisions the same architecture is maintained, with the new spindles (orange) in tangential directions, far from the animal-vegetal axis on which spermatozoon mitochondria (M) where arranged (light green circle). Nuclei in dark green.

Microtubule staining with anti-α-tubulin antibody in early embryos showed a higher density of short microtubules along the blastomere cortex and longer microtubules between cortex and nuclei, underlining the structural function of microtubular cytoskeleton in positioning the nucleus at the center of the cell (Figure 9). Furthermore, microtubule staining showed a well-defined midbody located in the middle of the cleavage furrow of 2-blastomere embryos (Figure 9D and E). In the same area the four mitochondria from spermatozoon were aggregated in male embryos, as shown by MitoTracker staining (Figure 9A; see also [62]). In 4-blastomere embryos the new spindles (marked by the two midbodies in Figure 9F and G) formed in tangential directions, around the region where the four M-type mitochondria were located, that was on the animal-vegetal (a–v) axis (Figure 9B, and scheme in Figure 10). The same could be seen in 8-blastomere embryos (Figure 9C and H).

**Figure 11:**
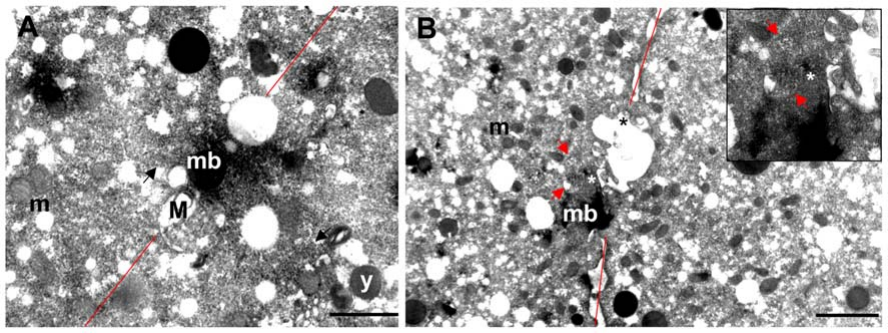
Ultrastructural analysis of *R. Philippinarum* male early embryos. (A, B) 2-blastomere embryos showing a midbody (mb). The cleavage plane is marked with a red line. (A) One spermatozoon mitochondrion of about 1 µm (M) is positioned near the midbody; black arrows indicate microtubules; m – egg mitochondria; y – yolk (bar 1 µm). (B) The egg mitochondria (m), abundant at the cleavage level, are recognizable by the smaller size; some are fusing with the cleavage membrane (black asterisk). Spermatozoon mitochondria (red arrows) are visible near the midbody (mb); a centriole (white asterisk) appears to still link the sperm mitochondria (detail in the inset) (bar 2 µm).

Finally, in some 2-blastomere embryos, TEM observations clearly showed a midbody associated to mitochondria of about 1 µm (Figure 11), which were most likely the M ones. Moreover, in some sections, a centriole appeared to still link spermatozoon mitochondria (Figure 11B). At the cleavage level, there was always an abundance of F-type mitochondria, clearly recognizable by their smaller size, some of which fusing with the cleavage membrane (Figure 11B).

## Discussion

### Unusual and conserved features of germ cells in DUI organisms

Germ line formation is a central topic to understand development. Recent researches [32]–[35] indicate that mitochondria play a key role in several steps of this process. For this reason, we used a DUI experimental system whose features provide a unique point of view for understanding the influence of mitochondria on germ line development, and we searched for typical structures involved in germ line specification. In *R. philippinarum* oogonia, TEM analysis showed electrondense granules that aggregate close to two mitochondrial masses at the opposite sides of the nucleus (Figure 2A). In oocytes these structures form two identical Bbs (Figure 2B and C) similar to those described in other animals. The presence of two distinct Bbs is an unusual feature; actually, as far as we know, *R. philippinarum* is the first species showing two Bbs in each oocyte, both containing a nuage clearly recognizable with TEM. This feature might be tentatively interpreted as a remnant of an archaic hermaphroditic condition [77]. In fact, hermaphrodite bivalves present an ovotestis with two separated parts: one producing eggs, the other producing sperm. Moreover, the presence of DUI in bivalves has been related to the maintenance of gonochorism [78]. More data relating the presence of two Bbs to hermaphroditism in Bivalvia are needed to investigate this hypothesis.

The Cb observed in *R. philippinarum* early spermatids (Figure 3) is morphologically identical to those described in mouse [20], [25] and rat [22], [23], [79]. It is always located close to the nuclear membrane and surrounded by empty mitochondria, some showing extruded cristae (Figure 3D), indicating their involvement in *R. philippinarum* Cb formation, as seen in sea urchins [33]. In the same cytoplasmic area, ribosomes with two different sizes and forming polyribosomes were found (Figure 3A–C), as reported for *Drosophila* [32]. Amikura et al. [35] demonstrated, by inhibition of prokaryotic-type translation, that the small ribosomes are of mitochondrial origin. The structural similarity between clams and model organisms suggests that these mitochondrial ribosomes play a similar role in the germ line development of this bivalve. In *R. philippinarum* spermatids, also the nucleus contributes to Cb formation, since material outflow from pores of the nuclear membrane can be seen in Cb proximity (Figure 3A–C), as documented in rats [79] and amphibians [80].

### Vasph and germ line formation

To detect Vasa expression, we used an antibody against Vasa chicken (anti-Cvh) [72]. This antibody reacted in monodimensional immunoblotting of gonadic and embryonic extracts with a band of about 65 kDa (Figure 4A) that with bidimensional immunoblotting is resolved in three spots (Figure 4B), probably isoforms of the same protein, with mass and isoelectric point in the range of the known Vasa-homologs [14]. Further, we compared the sequence of *R. philippinarum* Vasa (Vasph) to chicken Vasa. The detection of one protein by western blot, the high CORE index of the alignment of Vasph and chicken Vasa-homolog (score = 95) (Figure 5) and the identification in Vasph of Vasa conserved domains by InterProScan (Figure 6) support the specific staining of the antibody used, even more considering the specific labeling observed in gametogenetic cells, while no staining was found in other tissues (intestine and connective) (Figure 7). Thus, Vasa appears to be a specific marker of germ cells in *R. philippinarum*, as in other animals (see [12]–[15], [81]–[85]). Up to now, only the distribution of *vasa* mRNAs was analyzed in molluscs [86]–[91], and this is the first study about the immunolocalization of Vasa protein. Clam gonad is not permanent and the mechanism of its reformation every mating season is not fully documented. We showed that the wall of the acini are formed by Vasa labeled cells (germ cells). This cell aggregation defines an empty area in the middle (Figure 7C–F), the acinus lumen, which in mature gonads is full of gametes (Figure 7G and H).

Vasa is an evolutionary conserved component of the germ plasm [12]–[19], so we used this molecular marker to localize germ plasm both in eggs and in early embryos of *R. philippinarum*. In eggs labeled with anti-Cvh, a spotted staining dispersed in the cytoplasm was found, while a more strongly labeled zone was visible along the cleavage furrow in 2-bastomere embryos (Figure 8). This finding is in line with what found in embryos of other animals, in which Vasa localized in the middle portion of the first cleavage furrow [72]. This early Vasa segregation supports a preformation mechanism for germ line determination in *R. philippinarum*. In animals, germ cells can be specified by maternally inherited determinants (preformation) and by inductive signals from surrounding tissues during development (epigenesis) [92]. Among bivalves, in *Crassostrea gigas* [87] and *Sphaerium striatinum* [93] *vasa* mRNA is restricted to a defined zone during the first embryo stages suggesting a specification by preformation. The early segregation of Vasa in *R. philippinarum* suggests that maternal factors already present in the eggs could be involved in PGCs formation, supporting the hypothesis that maternal nuclear genes act as germ line determining factors (see also [68]).

### Spermatozoon mitochondria and male germ line formation

As discussed, both nuclear and mitochondrial factors play a role in nuage formation in both SMI and DUI species. However, a few questions remain open in DUI species. How do sperm mitochondria enter male germ line? How do they displace the maternal mitochondria in the germ cells? In *M. galloprovincialis* sperm mitochondria and nucleus remain at the entry point of eggs treated with colchicine [49], suggesting a role of microtubules in the movements of sperm mitochondria soon after fertilization. In *R. philippinarum* embryos, the microtubule immunostaining showed a well-defined midbody in the middle of the first cleavage furrow, corresponding to the area in which the aggregate of spermatozoon mitochondria localizes (Figure 9). It is known that when the cleavage furrow ingresses compressing the spindle midzone, it creates an intercellular bridge containing a midbody at the center [94]–[96]. Thus, the midbody is a structure of tightly packed microtubules and associated proteins derived from the constriction of the central spindle [96]. Although no clear function has been ascribed to the midbody yet, it contains proteins indispensable for cytokinesis, asymmetric cell division, and chromosome segregation [95]–[97]. It might be also involved in the trafficking of important signaling molecules along the microtubules and in the segregation of material for germ line development. Indeed, the germ plasm is segregated into germ cells by dynein- and kinesin-dependent transport on centrosome-nucleated microtubules [8], [98], and the disruption of some midbody proteins causes a failure in progeny production [95], [99]–[101]. In this way, in many animals germ line determinants aggregate in the middle of the first cleavage furrow [72], [102], where in DUI male embryos the aggregate of spermatozoon mitochondria localizes. In this context, M-type mitochondria may be recognized by specific motor proteins that would carry them to the central spindle, where the cleavage furrow of the 2-cell stage forms. The midbody appears to have a role in positioning M-type mitochondria in a stable zygote area on the a-v axis, avoiding a complete dispersal in the blastomeres: in spiral segmentation this central zone is not directly involved in the cleavages, which take place around it [103], so that the mitochondrial aggregation can be maintained. As clearly shown in 4-blastomere embryos of *R. philippinarum*, the new spindles form in tangential directions, far from the region on the a-v axis where the aggregate of M-type mitochondria is located (Figures 9 and 10). Although microtubules emanate from the spindle poles during early anaphase, the microtubules of the central spindle eventually lose their connections with poles [104], and any material localized in the midzone can be easily maintained in position. TEM observations on sections of *R. philippinarum* 2-blastomere embryos showed F-type mitochondria at the division plane, some fused with the cleavage membrane (Figure 11), supporting their involvement in its building, as already proposed ([95], [105] and references therein). Moreover, the same sections showed a midbody in proximity of one or more mitochondria of about 1 µm in diameter (Figure 11). These should be interpreted as spermatozoon mitochondria, since no organelle of a comparable size was found in unfertilized oocytes. In some sections a complete spermatozoon midpiece was detected and a centriole appeared to still link the M-type mitochondria (Figure 11B). On the basis of our observations, in male embryos of *R. philippinarum* sperm mitochondria are not detached from the distal centriole of the midpiece, allowing simple transport to the midzone by microtubule nucleation. In mammals, the sperm mitochondrial sheath appears to be associated with microtubule-based structures to secure the destruction of sperm mitochondria in a specific compartment of the embryo [106]. In DUI species a similar mechanism could be responsible for the transportation of male mitochondria towards PGCs.

Recent studies have addressed the question about which mitochondria enter the germ line, i.e. whether they are a random sub-sample or a selected small group, supporting the presence of a specific group of mitochondria in the germ plasm and their origin from the Bb [7], [10], [21], [34]. This condition appears to be a conserved feature in SMI animals. However, it is unclear how these mitochondria are selected among the mitochondrial population of the egg. Some papers suggested they could be the healthiest mitochondria for a better embryo development [10], [21], [47], [107], [108]. In DUI species two types of mitochondria with highly divergent genomes are differently transmitted, indicating that only genomes with specific features must selectively enter the germ line [63], [64]. During the development of DUI male embryos, spermatozoon mitochondria must be recognized by egg factors to be actively transferred by microtubules together with germ plasm in the PGCs, where they become dominant and replace Bb mitochondria during germ line formation.
